# Arsenic in drinking water and cerebrovascular disease, diabetes mellitus, and kidney disease in Michigan: a standardized mortality ratio analysis

**DOI:** 10.1186/1476-069X-6-4

**Published:** 2007-02-02

**Authors:** Jaymie R Meliker, Robert L Wahl, Lorraine L Cameron, Jerome O Nriagu

**Affiliations:** 1 BioMedware, Inc., Ann Arbor, Michigan, USA; 2 Division of Environmental and Occupational Epidemiology, Michigan Department of Community Health, Lansing, Michigan, USA; 3 Department of Environmental Health Sciences, School of Public Health, the University of Michigan, Ann Arbor, Michigan, USA

## Abstract

**Background:**

Exposure to arsenic concentrations in drinking water in excess of 300 μg/L is associated with diseases of the circulatory and respiratory system, several types of cancer, and diabetes; however, little is known about the health consequences of exposure to low-to-moderate levels of arsenic (10–100 μg/L).

**Methods:**

A standardized mortality ratio (SMR) analysis was conducted in a contiguous six county study area of southeastern Michigan to investigate the relationship between moderate arsenic levels and twenty-three selected disease outcomes. Disease outcomes included several types of cancer, diseases of the circulatory and respiratory system, diabetes mellitus, and kidney and liver diseases. Arsenic data were compiled from 9251 well water samples tested by the Michigan Department of Environmental Quality from 1983 through 2002. Michigan Resident Death Files data were amassed for 1979 through 1997 and sex-specific SMR analyses were conducted with indirect adjustment for age and race; 99% confidence intervals (CI) were reported.

**Results:**

The six county study area had a population-weighted mean arsenic concentration of 11.00 μg/L and a population-weighted median of 7.58 μg/L. SMR analyses were conducted for the entire six county study area, for only Genesee County (the most populous and urban county), and for the five counties besides Genesee. Concordance of results across analyses is used to interpret the findings. Elevated mortality rates were observed for both males (M) and females (F) for all diseases of the circulatory system (M SMR, 1.11; CI, 1.09–1.13; F SMR, 1.15; CI, 1.13,-1.17), cerebrovascular diseases (M SMR, 1.19; CI, 1.14–1.25; F SMR, 1.19; CI, 1.15–1.23), diabetes mellitus (M SMR, 1.28; CI, 1.18–1.37; F SMR, 1.27; CI, 1.19–1.35), and kidney diseases (M SMR, 1.28; CI, 1.15–1.42; F SMR, 1.38; CI, 1.25–1.52).

**Conclusion:**

This is some of the first evidence to suggest that exposure to low-to-moderate levels of arsenic in drinking water may be associated with several of the leading causes of mortality, although further epidemiologic studies are required to confirm the results suggested by this ecologic SMR analysis.

## Background

Assessment of health risks associated with exposure to moderately elevated levels of arsenic in drinking water (10–100 μg/l) has become the subject of considerable interest and some controversy in both regulatory and public health communities. The National Research Council (NRC) subcommittee on Arsenic in Drinking Water, for instance, found that "additional epidemiological evaluations are needed to characterize the dose-response relationship for arsenic-associated cancer and noncancer end points, especially at low doses" [1](see page 3) and simultaneously concluded that the guideline of "50 μg/L does not achieve...public health protection, and therefore, requires downward revision as promptly as possible." [1](see page 9). In the end, the United States Environmental Protection Agency (USEPA) recommended a reduction in the maximum contaminant level (MCL) to 10 μg/l for arsenic in US public drinking water supplies [2].

The call for a significant reduction in the MCL by the USEPA was prompted, at least in part, by findings of internal cancers (especially bladder, kidney, liver, and lung) among populations in Taiwan, Japan, Chile, and Argentina that are exposed to elevated levels of arsenic (typically > 300 μg/l) in their drinking water [3-8]. In addition to cancer, ample evidence exists to support a relationship between arsenic in drinking water and cardiovascular and circulatory diseases such as blackfoot disease [9,10], ischemic heart diseases [11], and cerebrovascular diseases [12]. Emerging evidence also suggests an association between arsenic and diabetes mellitus [13,14] and nonmalignant respiratory diseases [15,16]. Most of these studies, however, examined arsenic concentrations of 100 μg/L and above, providing little insight into health effects from low-to-moderate concentrations (10–100 μg/L) which are more commonly found in sources of drinking water in the US and Europe.

A few mortality studies have been conducted in areas where arsenic concentrations in drinking water are commonly in the 10–100 μg/L range; however, a clear picture of the relevant health risks has not yet emerged. Engel and Smith (1994) conducted a standardized mortality ratio (SMR) analysis for vascular and respiratory diseases in thirty US counties with elevated levels of arsenic in drinking water. Diseases of arteries, arterioles, and capillaries (DAAC), emphysema, and chronic airways obstruction exhibited significantly elevated SMRs in counties where mean arsenic levels exceeded 20 μg/L. In a cohort mortality study in Millard County, Utah where arsenic levels ranged from 14–166 μg/L, Lewis et al. [17] examined cancer and a host of cardiovascular, respiratory, and kidney diseases. These authors reported significant positive SMRs for women and men from hypertensive heart diseases, and for only men from nephritis and nephrosis, and prostate cancer. In contrast to the results from Engel and Smith [10], however, Lewis et al [17] did not identify an elevated SMR for DAAC, and did not investigate deaths caused by emphysema or chronic airways obstruction. In ecologic studies conducted in Belgium [18] and Hungary [19], low-to-moderate levels of arsenic were not reported to be associated with mortality due to diseases of the nervous system, circulatory system, liver, or cancer. Individual-level incidence studies of low-to-moderate arsenic exposure have also generated ambiguous findings with regard to the role of arsenic in cancers of the bladder and skin [20-25].

In light of this uncertainty, it is important to continue to investigate health risks from exposure to arsenic concentrations in the 10–100 μg/L range. Therefore, the goal of this study is to investigate mortality rates for twenty-three different health outcomes, including several types of cancer, circulatory and respiratory diseases, diabetes, and diseases of the kidneys and liver in six contiguous counties of Michigan with moderately elevated levels of arsenic in drinking water.

## Methods

### Arsenic in southeastern Michigan study area

**Figure 1:**
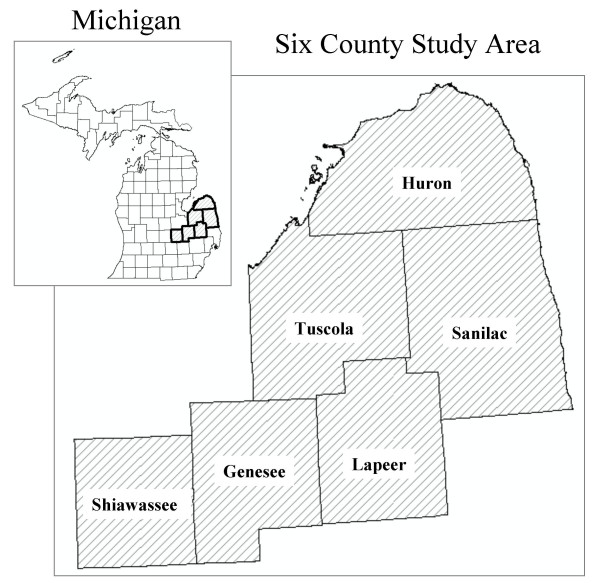
The six-county study area in the "thumb" region of southeastern Michigan.

Elevated concentrations of arsenic in Michigan groundwater were first reported from a well supplying a mobile home park in Huron County in 1981 [26]. Since then, arsenic has been identified in groundwater supplies, typically in the 10–100 μg/L range, throughout six contiguous counties of Michigan and is now a well-documented phenomenon that has generated considerable concern [27-29]. The counties involved include Genesee, Huron, Lapeer, Sanilac, Shiawassee, and Tuscola, and are located in the Michigan thumb region (Figure 1). The 2000 US Census indicates the six counties have a population of approximately 740,000 people and occupy an area of approximately 11,500 km^2^. The majority of the population resides in Genesee County (439,000), while the remainder is split fairly evenly among the other five counties (Table 1). Genesee County also contains the industrial city of Flint and the largest proportion of African Americans in the study area; in contrast, the other five counties are predominantly rural and white.

**Table 1:** Exposure and Population Statistics for Counties in Southeastern Michigan Study Area

	Genesee County	Huron County	Lapeer County	Sanilac County	Shiawassee County	Tuscola County	Six Counties Combined	Remainder of Michigan
Number of Groundwater Samples Analyzed	2,768	2,068	2,419	729	822	445	9,251	23,691
% of Population Drinking Groundwater	50%	84%	82%	95%	100%	100%	68%	44%
Population-weighted Mean Arsenic Concentration (μg/L)	10.42	12.21	19.26	9.97	6.66	8.08	11.00	2.98
Population-weighted Median Arsenic Concentration (μg/L)	8.48	3.50	11.98	4.61	3.91	3.32	7.58	1.27
Population Size	430,459	34,951	74,768	39,928	69,770	55,498	705,383	8,589,914
White Race (%)	78%	99%	98%	98%	98%	97%	86%	74%
Residing in a Rural Area (%)	21%	90%	86%	100%	66%	88%	44%	30%
Lived in Same County 5 Years Earlier (%)	90%	89%	78%	82%	84%	85%	87%	83%
Population (above age 25) with at Least a Bachelor's Degree	13%	9%	9%	8%	10%	8%	11%	17%
Persons Below Poverty Level (%)	16%	15%	8%	14%	11%	13%	14%	13%
Median Household Income	$31,030	$21,852	$35,874	$23,107	$30,283	$27,374	$30,332	$31,020

Arsenic data collected from 1983–2002, provided by Michigan Department of Environmental Quality.

US Maximum Contaminant Limit for public drinking water supplies is 10 μg/L.

Population statistics from US Census, 1990.

Estimates of arsenic concentrations in drinking water in the six-county study area were compiled from the Michigan Department of Environmental Quality (MDEQ) arsenic database which contains results from water samples collected and analyzed between 1983 and 2002. The database includes 9,251 analyses of water samples from the six-county study area, with another 23,691 analyses conducted throughout the remainder of the state. This database is comprised of samples analyzed using various methodologies including graphite furnace atomic absorption spectrometry (GF/AAS) (1983–1987, 1989–1995), inductively coupled plasma (ICP) optical emission hydride generation (1987–1988), hydride flame (quartz tube AAS) (1989–1995), and ICP/mass spectrometry (1996-present); previous analyses have indicated that samples analyzed using different methods were highly correlated [30]. Approximately 86% of the analyses were of private wells, and 14% came from municipal wells. Analyses of water samples from private wells were performed at the request of property owners.

Using the MDEQ arsenic database, county-level mean and median arsenic concentrations were calculated for private wells. In addition, arsenic estimates were compiled for each public well water supply in the area from the MDEQ arsenic database [30]; those not drinking groundwater were served by municipal surface water from the Great Lakes which contains arsenic concentrations averaging 0.30 μg/L [31]. The size of the population served by private well, public well, and public surface water supplies was compiled from an MDEQ database and population-weighted estimates were tabulated.

### Mortality and population data

Cause-specific mortalities from 1979 to 1997 were compiled from Michigan Resident Death Files by the Vital Records and Health Data Development Section of the Michigan Department of Community Health (MDCH). Twenty-three different underlying causes of death were included in the study, categorized according to the International Classification of Diseases, 9^th ^Revision (ICD-9). The twenty-three causes of death include several types of cancer, diseases of the circulatory and respiratory system, diabetes mellitus, and kidney and liver diseases, and are listed in Table 2. Each of these diseases has shown some evidence of association with arsenic exposure, although at higher concentrations in drinking water. The number of deaths from 1979 through 1997 by cause in each individual county and in the entire State were stratified by sex, race, and age and grouped into five-year categories beginning at age 35 and ending at ≥ 85 years of age. Census data and population estimates were compiled at five-year time intervals from the same period for the State of Michigan and the six counties in the study area.

**Table 2:** Standardized Mortality Ratios in Six County Southeastern Michigan Study Area, 1979–1997.

Cause of Death	ICD-9	Males			Females		
		
		Obs	SMR	99% CI	Obs	SMR	99% CI
Cancers							
Colon and rectum	153–154	1,439	1.04	0.97–1.11	1,369	1.03	0.96–1.10
Liver and biliary Passages	155–156	260	0.85	0.72–1.00	300	1.04	0.89–1.20
Trachea, bronchus and lung	162	4,425	1.02	0.98–1.06	2,297	1.02	0.96–1.07
Skin melanoma	172	143	0.99	0.79–1.22	91	0.97	0.73–1.27
Other skin cancer	173	59	1.24	0.86–1.72	26	1.06	0.60–1.72
Female reproductive organs	179–184				1,300	1.11*	1.03–1.19
Prostate	185	1,448	1.03	0.96–1.10			
Bladder	188	348	0.94	0.82–1.08	171	0.98	0.80–1.19
Kidney and other urinary organs	189	325	1.06	0.91–1.22	194	1.00	0.82–1.20
Circulatory diseases							
All diseases of the circulatory system	390–459	25,907	1.11*	1.09–1.13	26,699	1.15*	1.13–1.17
Essential hypertension and hypertensive renal disease	401, 403	203	1.16	0.96–1.38	250	1.01	0.85–1.18
Hypertensive heart disease and hypertensive heart and renal disease	402, 404	398	0.88	0.77–1.00	612	1.01	0.91–1.12
Ischemic heart diseases	410–414	14,073	1.01	0.99–1.03	12,573	1.01	0.98–1.03
Cerebrovascular diseases	430–438	3,493	1.19*	1.14–1.25	5,010	1.19*	1.15–1.23
Diseases of arteries arterioles and capillaries (DAAC)	440–448	1,220	1.01	0.93–1.08	1,329	1.04	0.97–1.11
Atherosclerosis	440	523	1.01	0.90–1.13	884	1.11*	1.02–1.21
Aortic aneurysm	441	504	0.95	0.85–1.07	212	0.81*	0.67–0.96
Respiratory diseases							
All diseases of the respiratory system	460–519	4,433	1.05*	1.01–1.09	3,568	1.03	0.98–1.07
Emphysema	492	632	1.00	0.90–1.11	363	0.98	0.85–1.12
Chronic airways Obstruction	496	1,706	1.16*	1.09–1.24	1,134	1.14*	1.05–1.23
Other diseases							
Diabetes mellitus	250	1,249	1.28*	1.18–1.37	1,612	1.27*	1.19–1.35
Chronic liver diseases and cirrhosis	571	632	0.67*	0.60–0.74	416	0.87*	0.77–0.99
Kidney diseases	580–589	614	1.28*	1.15–1.42	679	1.38*	1.25–1.52

ICD-9 = International Classification of Diseases, 9^th ^Revision; Obs = Number of observed deaths; CI = confidence interval; *p < 0.01.

### Data analysis

Sex-specific SMRs were calculated by dividing the number of observed deaths from a given cause by the age- and race-adjusted expected values for Michigan [32]. Expected values were calculated using indirect adjustment; statewide mortality rates for each underlying cause, stratified by age, race, and sex were multiplied by the combined sex-, race-, and age-specific person-years in each county. To account for the large number of calculations (23 SMRs each for males and females), 99% confidence intervals, instead of 95%, were calculated. SMR analyses were conducted for the entire six county study area, for only Genesee County (the most populous, urban, and racially diverse county), and for the five counties besides Genesee. Concordance of results across analyses was used to interpret the findings.

## Results

The six county study area contains a population-weighted mean arsenic concentration of 11.00 μg/L and a population-weighted median of 7.58 μg/L. In comparison, in the remainder of Michigan, the population-weighted mean is 2.98 μg/L with a median of 1.27 μg/L (Table 1).

SMRs are reported for twenty-three causes of death for males and females in the six county study area of southeastern Michigan (Table 2). Observed deaths from cancers were not different from the expected values for males or females, with the exception of cancer of the female reproductive organs (ICD-9 179–184), which was elevated. Deaths from all diseases of the circulatory system (ICD-9 390–459) and cerebrovascular diseases (ICD-9 430–438) were elevated in both males and females. In females only, deaths from atherosclerosis (ICD-9 440) were elevated and deaths from aortic aneurysm (ICD-9 441) were reduced. Deaths from respiratory diseases were elevated in the study area, with chronic airways obstruction-related deaths elevated in both males and females (ICD-9 496). Males also experienced elevated mortality rates for all diseases of the respiratory system (ICD-9 460–519). Deaths from diabetes mellitus (ICD-9 250) and kidney diseases (ICD-9 580–589) were also elevated among both males and females in the study area. Deaths from chronic liver diseases and cirrhosis (ICD-9 571) were reduced for males and females.

In Genesee County alone, mortality rates were elevated for the same health outcomes as those identified from the six county analysis (Table 3). In addition, elevated mortality rates were observed for both males and females from trachea, bronchus, and lung cancer (ICD-9 162), ischemic heart diseases (ICD-9 410–414), and all diseases of the respiratory system (ICD-9 460–519). Deaths were also elevated for only males from non-melanoma skin cancer (ICD-9 173) and essential hypertension and hypertensive renal disease (ICD-9 401, 403). Deaths from chronic liver diseases and cirrhosis (ICD-9 571) were no longer significantly reduced for females.

**Table 3:** Standardized Mortality Ratios in Genesee County, Michigan, 1979–1997.

Cause of Death	ICD-9	Males			Females		
		
		Obs	SMR	99% CI	Obs	SMR	99% CI
Cancers							
Colon and rectum	153–154	814	1.04	0.95–1.14	800	1.02	0.93–1.11
Liver and biliary Passages	155–156	150	0.84	0.67–1.03	171	1.00	0.82–1.22
Trachea, bronchus and lung	162	2,763	1.10*	1.04–1.15	1,526	1.13*	1.06–1.21
Skin melanoma	172	85	1.07	0.80–1.41	48	0.90	0.60–1.30
Other skin cancer	173	43	1.61*	1.05–2.36	11	0.76	0.30–1.58
Female reproductive organs	179–184				804	1.15*	1.05–1.26
Prostate	185	787	0.98	0.90–1.08			
Bladder	188	209	1.05	0.87–1.25	101	0.99	0.75–1.27
Kidney and other urinary organs	189	182	1.04	0.85–1.26	119	1.04	0.81–1.31
Circulatory diseases							
All diseases of the circulatory system	390–459	15,033	1.14*	1.12–1.17	15,863	1.16*	1.14–1.19
Essential hypertension and hypertensive renal disease	401, 403	139	1.31*	1.04–1.63	160	1.04	0.84–1.27
Hypertensive heart disease and hypertensive heart and renal disease	402, 404	311	1.07	0.92–1.24	427	1.12	0.99–1.27
Ischemic heart Diseases	410–414	7,981	1.05*	1.02–1.08	7,589	1.05*	1.02–1.08
Cerebrovascular diseases	430–438	1,893	1.15*	1.08–1.22	2,815	1.14*	1.09–1.20
Diseases of arteries arterioles and capillaries (DAAC)	440–448	669	1.02	0.92–1.12	760	1.04	0.95–1.14
Atherosclerosis	440	300	1.09	0.93–1.26	510	1.14*	1.01–1.27
Aortic aneurysm	441	257	0.89	0.76–1.05	115	0.75*	0.58–0.95
Respiratory diseases							
All diseases of the respiratory system	460–519	2,522	1.08*	1.03–1.14	2,235	1.11*	1.05–1.17
Emphysema	492	373	1.09	0.95–1.25	250	1.18	1.00–1.39
Chronic airways Obstruction	496	985	1.23*	1.14–1.34	725	1.27*	1.16–1.40
Other diseases							
Diabetes mellitus	250	737	1.30*	1.18–1.43	979	1.28*	1.17–1.38
Chronic liver diseases and cirrhosis	571	343	0.58*	0.50–0.66	268	0.89	0.75–1.03
Kidney diseases	580–589	335	1.21*	1.04–1.39	418	1.40*	1.23–1.58

ICD-9 = International Classification of Diseases, 9^th ^Revision; Obs = Number of observed deaths; CI = confidence interval; *p < 0.01.

In the five counties other than Genesee County, mortality rates remained elevated for males and females from all diseases of the circulatory system (ICD-9 390–459), cerebrovascular diseases (ICD-9 430–438), diabetes mellitus (ICD-9 250), and kidney diseases (ICD-9 580–589) (Table 4). Deaths from chronic airways obstruction (ICD-9 496) were no longer elevated. In fact, mortality rates were significantly reduced for females from all diseases of the respiratory system (ICD-9 460–519) and emphysema (ICD-9 492). Deaths were also reduced for males and females from trachea, bronchus, and lung cancer (ICD-9 162) and hypertensive heart disease and hypertensive heart and renal disease (ICD-9 402, 404), for females from ischemic heart diseases (ICD-9 410–414), and for males from chronic liver diseases and cirrhosis (ICD-9 571).

**Table 4:** Standardized Mortality Ratios in Five Counties of Study Area Excluding Genesee County, Michigan, 1979–1997.

Cause of Death	ICD-9	Males			Females		
		
		Obs	SMR	99% CI	Obs	SMR	99% CI
Cancers							
Colon and rectum	153–154	625	1.04	0.93–1.15	569	1.05	0.94–1.17
Liver and biliary Passages	155–156	111	0.88	0.68–1.12	129	1.09	0.86–1.36
Trachea, bronchus and lung	162	1,662	0.91*	0.85–0.97	771	0.85*	0.77–0.93
Skin melanoma	172	58	0.88	0.61–1.23	43	1.06	0.69–1.56
Other skin cancer	173	16	0.77	0.36–1.41	15	1.47	0.67–2.76
Female reproductive organs	179–184				496	1.05	0.93–1.17
Prostate	185	661	1.08	0.98–1.20			
Bladder	188	139	0.82	0.65–1.02	70	0.97	0.70–1.31
Kidney and other urinary organs	189	143	1.07	0.86–1.33	75	0.93	0.68–1.25
Circulatory diseases							
All diseases of the circulatory system	390–459	10,874	1.06*	1.04–1.09	10,836	1.12*	1.10–1.15
Essential hypertension and hypertensive renal disease	401, 403	64	0.92	0.65–1.26	90	0.95	0.71–1.24
Hypertensive heart disease and hypertensive heart and renal disease	402, 404	87	0.53*	0.40–0.70	185	0.82*	0.67–0.98
Ischemic heart diseases	410–414	6,092	0.97	0.94–1.00	4,984	0.94*	0.91–0.98
Cerebrovascular diseases	430–438	1,600	1.25*	1.17–1.33	2,195	1.26*	1.19–1.33
Diseases of arteries arterioles and capillaries (DAAC)	440–448	551	0.99	0.88–1.10	569	1.04	0.93–1.16
Atherosclerosis	440	223	0.92	0.77–1.09	374	1.08	0.94–1.23
Aortic aneurysm	441	247	1.02	0.86–1.20	97	0.88	0.67–1.14
Respiratory diseases							
All diseases of the respiratory system	460–519	1,911	1.01	0.95–1.07	1,333	0.91*	0.85–0.98
Emphysema	492	259	0.89	0.75–1.04	113	0.71*	0.55–0.90
Chronic airways Obstruction	496	721	1.08	0.98–1.19	409	0.96	0.84–1.09
Other diseases							
Diabetes mellitus	250	512	1.24*	1.10–1.39	633	1.26*	1.14–1.40
Chronic liver diseases and cirrhosis	571	289	0.82*	0.70–0.96	148	0.86	0.68–1.05
Kidney diseases	580–589	279	1.37*	1.16–1.59	261	1.35*	1.15–1.58

ICD-9 = International Classification of Diseases, 9^th ^Revision; Obs = Number of observed deaths; CI = confidence interval; *p < 0.01.

## Discussion

In these six county, five county, and Genesee county-only SMR analyses of the Michigan thumb region, heightened mortality rates were consistently observed for diabetes mellitus, cerebrovascular diseases, and kidney diseases in both males and females. Each of these health endpoints is reported to be associated with arsenic in drinking water at levels in excess of 200–300 μg/L; here we present some of the first evidence of a relationship between these health outcomes and arsenic concentrations in a region of the US where elevated concentrations typically range from 10–100 μg/L.

Arsenic has been shown to be associated with increased rates of diabetes incidence [14], prevalence [13,33], and mortality [8] in studies from Taiwan and Bangladesh. Only one previous study, however, examined risk of mortality from diabetes from drinking water arsenic concentrations below 200 μg/L and that study found no association [17]. In contrast, we report elevated mortality rates for diabetes in both males (SMR, 1.28; 99%CI, 1.18–1.37) and females (SMR, 1.27; 99%CI, 1.19–1.35) (Table 2). This inconsistency may be explained by the added power in our study which includes 1249 male and 1612 female deaths from diabetes, compared with only 20 male and 35 female deaths in the Utah study by Lewis et al. [17]. In addition, the Utah study relied on data from a cohort of members of the Church of Jesus Christ of Latter-day Saints whose personal lifestyle choices (e.g., no tobacco smoking or consumption of alcohol) make comparisons with other study populations difficult. Diabetes mellitus is a complex disease, defined as a set of abnormalities characterized by a state of sustained hyperglycemia. It is the sixth leading cause of death in the United States, with unknown specific etiology [34,35]. The features of diabetes mellitus most commonly observed in arsenic-exposed individuals are similar to non-insulin-dependent diabetes mellitus or Type 2 diabetes [14]. Although the biological mechanisms responsible for arsenic-induced diabetes mellitus are largely unknown, recent evidence suggests that the trivalent arsenicals may suppress insulin-stimulated glucose uptake by interfering with mobilization of glucose transporters in adipose cells [36], as well as interfering with transcription factors involved in insulin-related gene expression [37].

Kidney diseases are the ninth leading cause of death in the United States [35] and were also found to be elevated in the six county study area for males (SMR, 1.28; 99%CI, 1.15–1.42) and females (SMR, 1.38; 99%CI, 1.25–1.52), as well as in the five county and Genesee County-only analyses. Elevated mortality rates for kidney diseases have been reported in high arsenic areas of Taiwan [8], and significantly elevated SMRs were also reported from nephritis and nephrosis in Utah men [17]. Few mechanisms of arsenic-induced kidney diseases have been proposed, however. Kidney diseases are a frequent complication of diabetes, and arsenic may be affecting the kidney via vascular changes associated with diabetes.

In addition to diabetes and kidney diseases, elevated mortality rates were observed for cerebrovascular diseases for males (SMR, 1.19; 99%CI, 1.14–1.25) and females (SMR, 1.19; 99%CI, 1.15–1.23). Elevated mortality rates were also observed when grouping together all diseases of the circulatory system, however, only mortality from cerebrovascular diseases was consistently elevated for both males and females when examining the individual major causes of vascular-related death. Cerebrovascular diseases are the third leading cause of death in the United States [35]. Epidemiologic studies have previously reported increased prevalence [12] and mortality [8] from cerebrovascular diseases in high arsenic areas of Taiwan. Chiou et al. [12] found prevalence of cerebrovascular diseases to be significantly associated with arsenic levels as low as 0.1–50 μg/L in drinking water (compared to a baseline of <0.1 μg/L). Increased mortality from cerebrovascular diseases, however, was not found in previous ecologic studies of health effects from low-to-moderate arsenic exposure in the US [10,17]. Experimental studies suggest potential mechanisms for cerebrovascular toxicity of arsenic include inflammatory and coagulatory activity of endothelial cells, increased oxidative stress, and impaired vascular nitric oxide homeostatis [38]. These studies, however, have typically been performed using unrealistically high arsenic concentrations calling into question their mechanistic relevance [39]. Our study lends support to the evidence that low-to-moderate levels of arsenic in drinking water are associated with elevated rates of cerebrovascular diseases.

Mixed results were observed for several other circulatory disease outcomes across the six county, five county, and Genesee County-only analyses. Deaths from atherosclerosis were significantly elevated and deaths from aortic aneurysm were significantly reduced for females in the six county and Genesee County-only analyses, but not in the five county analyses. Deaths from ischaemic heart diseases (for males and females) and essential hypertension and hypertensive renal disease (for males only) were elevated only in the Genesee County-only analyses. In only the five county analyses, deaths from hypertensive heart disease and hypertensive heart and renal disease were reduced for males and females. Taken as a whole, these results suggest that, with the exception of cerebrovascular diseases, deaths from different types of circulatory diseases were elevated in Genesee County but not in the other five counties.

Differences in cancer and respiratory outcomes were also detected between Genesee County and the other five counties. In Genesee county, elevated mortality rates were observed for trachea, bronchus, and lung cancer (for males and females), other skin cancer (for males), cancer of the female reproductive organs, all diseases of the respiratory system (for males and females), and chronic airways obstruction (for males and females). None of these disease outcomes were significantly elevated in the five county analyses, and mortality rates were significantly reduced for cancers of the trachea, bronchus, and lung (for males and females), emphysema (for females), and all diseases of the respiratory system (for females).

These differences in mortality profiles between Genesee County and the other five counties may reflect differences commonly observed between urban and rural residents. Approximately 21% of the population in Genesee county resides in rural areas, compared with more than 80% in the other five counties (Table 1). Farmers and rural residents have been shown to have lower mortality due to all causes combined, all cancers combined, respiratory system cancer, cardiovascular disease, and respiratory diseases other than cancer compared to non-farmers and urban residents [40-44]. A lower prevalence of smoking among farmers compared with non-farmers is often cited as one explanation for the lower mortality among the former population [41-43]. A lower smoking prevalence among our study population may also be a factor suppressing mortality within the five county area, especially since smoking is a key risk factor for many of the diseases in question. Although data were not available on historical smoking prevalence for this population or for Michigan, a comparison of data from the 1976 National Health Interview Survey with a previous cohort study conducted in the region in the 1970s indicated that proportionally fewer adults in the region were smokers (25.1%) compared with the adult white national population (36.0%) [45,46]. These differences suggest that results which appear to be consistent across the six county, five county, and Genesee-county analyses are likely to be the least confounded.

Aside from potential differences in historical smoking behaviors among the rural segment of the population, additional differences between the six county study area and the remainder of the state of Michigan deserve comment (Table 5). Michigan Behavioral Risk Factor Survey (BRFS) data from 1989–1993 indicate lower age-adjusted rates of heavy or binge drinking from this part of Michigan, as compared to the state as a whole [47]. This may explain the reduced mortality rates observed for chronic liver diseases and cirrhosis, which were consistent for males across the different analyses.  Nevertheless, it is possible that low-to-moderate levels of arsenic in drinking water are protective against liver diseases, although this would be surprising since arsenic is a well-established risk factor for liver diseases when high doses are given in animal studies [48].

**Table 5:** Prevalence of Selected Behavioral Characteristics for Six County Study Area and Entire State of Michigan.

	Six County Study Area	Entire State of Michigan
Current smoking	25.1%	25.1%
Heavy drinking (60+ drinks/mo)	2.6%	3.7%
Binge drinking (5+ drinks on 1+ occasions in past month)	7.6%	11.7%
Cholesterol never checked	16.1%	19.3%
Ever told cholesterol high (among tested)	37.4%	33.0%
Ever told high blood pressure	33.6%	31.4%
Overweight (BMI >= 27.8 for men, >= 27.3 for women)	34.6%	33.4%
Never had mammogram (among women 40+)	19.0%	22.2%

From Michigan Behavioral Risk Factor Survey, 1989–1993; age-adjusted.

The proportion of the population that lived in the same county five years earlier was slightly higher in the six county study area (87%) compared with the remainder of the state (83%) (Table 1). The six county study area also is considerably more rural than the State as a whole (Table 1). BRFS data generally indicate poorer access to health care in the most rural parts of the State [47], however higher rates of mammography and cholesterol screening were reported in this part of Michigan compared with the State average (Table 5). It is unknown how these rates relate to screening for other diseases. A slightly higher prevalence of cardiovascular disease risk factors such as obesity, high blood pressure, and high cholesterol has also been reported in the study area. Smoking prevalence in the study area in the early 1990s, however, is no different than that of the state as a whole. Obesity, high blood pressure, cigarette smoking, and poor access to health care are established risk factors for the diseases of strongest significance here [49,50], however not all of these factors are elevated throughout the study area. In addition, high blood pressure may not be a confounder, but rather could be an intermediate variable in cerebrovascular mortality. Results indicate that deaths due to diseases strongly associated with these risk factors, such as emphysema, lung cancer, ischemic heart diseases, and DAAC were not elevated throughout our study area, suggesting that smoking, poor access to health care, obesity, and high blood pressure can only partially explain the elevated mortality rates observed for kidney and cerebrovascular diseases and diabetes mellitus.

In light of the attention given to arsenic and cancer [3-7], the lack of findings in our study of significantly elevated mortality rates for cancers of the bladder, kidney, lung, and skin is intriguing. The SMR for lung cancer was elevated in Genesee County, but depressed in the other five counties. This may be due to a possible synergism between arsenic and smoking [51], as Genesee is the most urban county, presumably with the highest smoking rates [41-43]. An alternative explanation for the lack of strong cancer-related findings may be that arsenic levels in groundwater of southeastern Michigan are below the threshold for cancer induction, or there may be moderating factors which were not considered here. However, if the excess risk for these cancers is small, it is possible that ecologic studies will be unable to detect significant risk, as was cautioned by the NRC subcommittee on Arsenic in Drinking Water [52](see page 223). In addition, the use of mortality rates may not be the best measure for certain cancer outcomes, such as skin and bladder cancer, which have relatively high survival rates [53].

In interpreting results of this ecologic analysis, other aspects of the study deserve consideration. This study did not assess the accuracy and precision of the arsenic laboratory measurements nor the proportion of each arsenic species (As(III) and As(V)) in the drinking water samples. Private wells were preferentially sampled based on homeowner requests, possibly causing an overestimate of population-based arsenic exposure, as has been shown in another region of the US [54]. This study also did not investigate differences in the reporting and classification of underlying causes of death across counties and regions. Such reporting and classification differences are common in mortality studies of diabetes; however, these differences are less common for mortality studies of kidney and cerebrovascular diseases [55].

Additional limitations characteristic of ecologic studies also need to be kept in mind when interpreting our results: individual-level exposure has not been assessed; mortality in one geographic area does not imply that a person lived there for long periods of his or her life; and confounding variables such as smoking and obesity were not included in the quantitative analyses. Since there is no individual-level exposure assessment, interpretation of exposure at the individual-level would result in the Berkson measurement error [52]. Furthermore, as is forewarned by the ecologic fallacy, conclusions should not be drawn at the individual-level because there was no individual-level assessment of the exposure-disease relationship, only a county-level assessment.

## Conclusion

These limitations not withstanding, this region of southeastern Michigan was selected because of moderately elevated concentrations of arsenic in groundwater, a large percentage of the population using groundwater as their drinking water source, and low rates of migration in and out of the study area [30]. Health risks from long-term ingestion of water containing arsenic concentrations in the 10–100 μg/L range are uncertain, and this ecologic study is a first step in suggesting that moderately elevated arsenic concentrations are associated with mortality from cerebrovascular diseases, diabetes mellitus, and kidney diseases. Carefully planned individual-level epidemiologic studies are necessary to further investigate this relationship.

## Abbreviations

AAS Atomic Absorption Spectrometry

BRFS Behavioral Risk Factor Survey

CI Confidence Interval

DAAC Diseases of Arteries, Arterioles, and Capillaries

EPA Environmental Protection Agency

GF Graphite Furnace

ICD-9 International Classification of Diseases, 9^th ^Revision

ICP Inductively Coupled Plasma

MCL Maximum Contaminant Level

MDCH Michigan Department of Community Health

MDEQ Michigan Department of Environmental Quality

SMR Standardized Mortality Ratio

## Competing interests

The author(s) declare that they have no competing interests.

## Authors' contributions

LC and RW conceived of and designed this study; RW gained access to the data and oversaw data coding and data management. JM conducted the analyses and drafted the manuscript. JN, LC, and RW offered analytical suggestions, assisted with interpretation, made critical revisions to the manuscript, and gave approval to the final draft.

## References

[B1] NRC (1999). Arsenic in Drinking Water.

[B2] USEPA (2001). National Primary Drinking Water Regulations: Arsenic and clarifications to compliance and new source contaminants monitoring; final rule Fed Reg.

[B3] Ferreccio C, Gonzalez C, Milosavjlevic V, Marshall G, Sancha AM, Smith AH (2000). Lung cancer and arsenic concentrations in drinking water in Chile. Epidemiology.

[B4] Guo H-R, Chiang H-S, Hu H, Lipsitz SR, Monson RR (1997). Arsenic in drinking water and incidence of urinary cancers. Epidemiology.

[B5] Hopenhayn-Rich C, Biggs ML, Fuchs A, Bergoglio R, Tello EE, Nicolli H, Smith AH (1996). Bladder cancer mortality associated with arsenic in drinking water in Argentina. Epidemiology.

[B6] Smith AH, Goycolea M, Haque R, Biggs ML (1998). Marked increase in bladder and lung cancer mortality in a region of Northern Chile due to arsenic in drinking water. Am J Epidemiol.

[B7] Steinmaus C, Moore L, Hopenhayn-Rich C, Biggs ML, Smith AH (2000). Arsenic in drinking water and bladder cancer. Cancer Invest.

[B8] Tsai S-M, Wang T-N, Ko Y-C (1999). Mortality for certain diseases in areas with high levels of arsenic in drinking water. Arch Environ Health.

[B9] Ch'i IC, Blackwell RQ (1968). A controlled retrospective study of blackfoot disease, an endemic peripheral gangrene disease in Taiwan. Am J Epidemiol.

[B10] Engel RR, Smith AH (1994). Arsenic in drinking water and mortality from vascular disease: an ecologic analysis in 30 counties in the United States. Arch Environ Health.

[B11] Chen C-J, Chiou H-Y, Chiang M-H, Lin L-J, Tai T-Y (1996). Dose-response relationship between ischemic heart disease mortality and long-term arsenic exposure. Arterioscl Throm Vas.

[B12] Chiou H-Y, Huang W-I, Su C-L, Chang S-F, Hsu Y-S, Chen C-J (1997). Dose-response relationship between prevalence of cerebrovascular disease and ingested inorganic arsenic. Stroke.

[B13] Rahman M, Tondel M, Ahmad SK, Axelson O (1998). Diabetes mellitus associated with arsenic exposure in Bangladesh. Am J Epidemiol.

[B14] Tseng C-H, Tai T-Y, Chong C-K, Tseng C-P, Lai M-S, Lin BJ, Chiou H-Y, Hsueh Y-M, Hsu K-H, Chen C-J (2000). Long-term arsenic exposure and incidence of non-insulin-dependent diabetes mellitus: A cohort study in arseniasis-hyperendemic villages in Taiwan. Environ Health Persp.

[B15] Mazumder DNG, Steinmaus C, Bhattacharya P, von Ehrenstein OS, Ghosh N, Gotway M, Sil A, Balmes JR, Haque R, Hira-Smith MM, Smith AH (2005). Bronchiectasis in persons with skin lesions resulting from arsenic in drinking water. Epidemiology.

[B16] Milton AH, Rahman M (2002). Respiratory effects and arsenic contaminated well water in Bangladesh. Int J Environ Health R.

[B17] Lewis DR, Southwick JW, Ouellet-Hellstrom R, Rench J, Calderon RL (1999). Drinking water arsenic in Utah: A cohort mortality study. Environ Health Persp.

[B18] Buchet JP, Lison D (1998). Mortality by cancer in groups of the Belgian population with a moderately increased intake of arsenic. Int Arch Occup Environ Health.

[B19] Varsanyi I, Fodre Z, Bartha A (1991). Arsenic in drinking water and mortality in the southern Great Plain, Hungary. Environ Geochem Hlth.

[B20] Bates MN, Smith AH, Cantor KP (1995). Case-control study of bladder cancer and arsenic in drinking water. Am J Epidemiol.

[B21] Bates MN, Rey OA, Biggs ML, Hopenhayn C, Moore LE, Kalman D, Steinmaus C, Smith AH (2004). Case-control study of bladder cancer and exposure to arsenic in drinking water in Argentina. Am J Epidemiol.

[B22] Karagas MR, Stukel TA, Morris JS, Tosteson TD, Weiss JE, Spencer SK, Greenberg ER (2001). Skin cancer risk in relation to toenail arsenic concentrations in a US population-based case-control study. Am J Epidemiol.

[B23] Karagas MR, Tosteson TD, Morris JS, Demidenko E, Mott LA, Heaney J, Schned A (2004). Incidence of transitional cell carcinoma of the bladder and arsenic exposure in New Hampshire. Cancer Cause Control.

[B24] Michaud DS, Wright ME, Cantor KP, Taylor PR, Virtamo J, Albanes D (2004). Arsenic concentrations in prediagnostic toenails and the risk of bladder cancer in a cohort study of male smokers. Am J Epidemiol.

[B25] Steinmaus C, Yuan Y, Bates MN, Smith AH (2003). Case-control study of bladder cancer and drinking water arsenic in the western United States. Am J Epidemiol.

[B26] Michigan Department of Public Health (1982). Arsenic in drinking water – A study of exposure and clinical survey.

[B27] Haack SK, Trecanni SL (2000). Arsenic concentration and selected geochemical characteristics for ground water and aquifer materials in southeastern Michigan.

[B28] Kim MJ, Nriagu JO, Haack S (2002). Arsenic species and chemistry in groundwater of southeast Michigan. Environ Pollut.

[B29] Kolker A, Haack SK, Cannon WF, Westjohn DB, Kim MJ, Nriagu J, Woodruff LG, Welch AH, Stollenwerk KG (2003). Arsenic in southeastern Michigan. Arsenic in Ground Water.

[B30] Meliker JR, Slotnick MJ, AvRuskin GA, Kaufmann A, Fedewa SA, Goovaerts P, Jacquez GM, Nriagu JO (2007). Individual lifetime exposure to inorganic arsenic using a Space-Time Information System. Int Arch Occup Environ Health.

[B31] Slotnick MJ, Meliker JR, Nriagu JO (2006). Effects of Time and Point-of-Use Devices on Arsenic Levels in Southeastern Michigan Drinking Water, USA. Sci Tot Environ.

[B32] Breslow NE, Day NE (1987). Statistical methods in cancer research Vol II: the design and analysis of cohort studies.

[B33] Lai MS, Hsueh YM, Chen CJ, Shyu MP, Chen SY, Kuo TL, Wu MM, Tai TY (1994). Ingested inorganic arsenic and prevalence of diabetes mellitus. Am J Epidemiol.

[B34] American Diabetics Association (2005). Standards of Medical Care in Diabetes. Diabetic Care.

[B35] Anderson RN, Smith BL (2005). Deaths: leading causes for 2002. National Vital Statistics Reports.

[B36] Walton FS, Harmon AW, Paul DS, Drobna Z, Patel YM, Styblo M (2004). Inhibition of insulin-dependent glucose uptake by trivalent arsenicals: possible mechanism of arsenic-induced diabetes. Toxicol Appl Pharmacol.

[B37] Salazard B, Bellon L, Jean S, Maraninchi M, El Yazidi C, Orsiere T, Margotat A, Botta A, Bergé-Lefranc J-L (2004). Low-level arsenite activates the transcription of genes involved in adipose differentiation. Cell Biol Toxicol.

[B38] Simeonova PP, Luster MI (2004). Arsenic and atherosclerosis. Toxicol Appl Pharmacol.

[B39] Navas-Acien A, Sharrett AR, Silbergeld EK, Schwartz BS, Nachman KE, Burke TA, Guallar E (2005). Arsenic exposure and cardiovascular disease: a systematic review of the epidemiological evidence. Am J Epidemiol.

[B40] Blair A, Dosemeci M, Heineman EF (1993). Cancer and other causes of death among male and female farmers from twenty-three states. Am J Ind Med.

[B41] Blair A, Sandler DP, Tarone R, Lubin J, Thomas K, Hoppin JA, Samanic C, Coble J, Kamel F, Knott C, Dosemeci M, Zahm SH, Lynch CF, Rothman N, Alavanja MCR (2005). Mortality among participants in the Agricultural Health Study. Ann Epidemiol.

[B42] Cerhan JR, Cantor KP, Williamson K, Lynch CF, Torner JC, Burmeister LF (1998). Cancer mortality among Iowa farmers: Recent results, time trends, and lifestyle factors (United States). Cancer Cause Control.

[B43] Folsom AR, Zhang S, Sellers TA, Zheng W, Kushi LH, Cerhan JR (1996). Cancer incidence among women living on farms: findings from the Iowa Women's Health Study. J Occup Environ Med.

[B44] Stiernstrom E, Holmberg S, Thelin A, Svarsudd K (2001). A prospective study of morbidity and mortality rates among farmers and rural and urban nonfarmers. J Clin Epidemiol.

[B45] U.S. Department of Health, Education and Welfare (1979). Smoking and Health: A Report of the Surgeon General.

[B46] Vasiliu O, Cameron L, Gardiner J, DeGuire P, Karmaus W (2006). Polybrominated biphenyls, polychlorinated biphenyls, body weight, and incidence of adult-onset diabetes mellitus. Epidemiology.

[B47] Schillo BA, Skarupski KA, McGee H, Rafferty A (1995). Michigan Risk Factor Surveillance System: Assessing Risk Factors at the Regional Level 1989–1993.

[B48] Liu T, Liu J, LeCluyse EL, Zhou YS, Cheng ML, Waalkes MP (2001). Application of cDNA microarray to the study of arsenic-induced liver diseases in the population of Guizhou, China. Toxicol Sci.

[B49] Paeratakul S, Lovejoy JC, Ryan DH, Bray GA (2002). The relation of gender, race and socioeconomic status to obesity and obesity comorbidities in a sample of US adults. Int J Obesity.

[B50] Sturm R (2002). The effects of obesity, smoking, and drinking on medical problems and costs. Health Affairs.

[B51] Chen CL, Hsu LI, Chiou HY, Hseuh YM, Chen SY, Wu MM, Chen CJ (2004). Ingested arsenic, cigarette smoking, and lung cancer risk. JAMA.

[B52] NRC (2001). Arsenic in Drinking Water: 2001 Update.

[B53] Adami H-O, Hunter D, Trichopoulos D (2002). Textbook of Cancer Epidemiology.

[B54] Peters SC, Blum JD, Klaue B, Karagas MR (1999). Arsenic occurrence in New Hampshire drinking water. Environ Sci Technol.

[B55] Morgan CL, Currie CJ, Peters JR (2000). Relationship between diabetes and mortality: a population study using record linkage. Diabetes Care.

